# Automatic Myotendinous Junction Tracking in Ultrasound Images with Phase-Based Segmentation

**DOI:** 10.1155/2018/3697835

**Published:** 2018-03-19

**Authors:** Guang-Quan Zhou, Yi Zhang, Ruo-Li Wang, Ping Zhou, Yong-Ping Zheng, Olga Tarassova, Anton Arndt, Qiang Chen

**Affiliations:** ^1^School of Biological Science and Medical Engineering, Southeast University, Nanjing, China; ^2^National Demonstration Center for Experimental Biomedical Engineering Education, Southeast University, Nanjing, China; ^3^Department of Women's and Children's Health, Karolinska Institute, Stockholm, Sweden; ^4^BioMEX Center and Department of Mechanics, Royal Institute of Technology, Stockholm, Sweden; ^5^Department of Biomedical Engineering, The Hong Kong Polytechnic University, Hung Hom, Hong Kong; ^6^The Swedish School of Sport and Health Sciences, Stockholm, Sweden; ^7^Department of Clinical Intervention and Technology, Karolinska Institute, Stockholm, Sweden

## Abstract

Displacement of the myotendinous junction (MTJ) obtained by ultrasound imaging is crucial to quantify the interactive length changes of muscles and tendons for understanding the mechanics and pathological conditions of the muscle-tendon unit during motion. However, the lack of a reliable automatic measurement method restricts its application in human motion analysis. This paper presents an automated measurement of MTJ displacement using prior knowledge on tendinous tissues and MTJ, precluding the influence of nontendinous components on the estimation of MTJ displacement. It is based on the perception of tendinous features from musculoskeletal ultrasound images using Radon transform and thresholding methods, with information about the symmetric measures obtained from phase congruency. The displacement of MTJ is achieved by tracking manually marked points on tendinous tissues with the Lucas-Kanade optical flow algorithm applied over the segmented MTJ region. The performance of this method was evaluated on ultrasound images of the gastrocnemius obtained from 10 healthy subjects (26.0 ± 2.9 years of age). Waveform similarity between the manual and automatic measurements was assessed by calculating the overall similarity with the coefficient of multiple correlation (CMC).* In vivo* experiments demonstrated that MTJ tracking with the proposed method (CMC = 0.97 ± 0.02) was more consistent with the manual measurements than existing optical flow tracking methods (CMC = 0.79 ± 0.11). This study demonstrated that the proposed method was robust to the interference of nontendinous components, resulting in a more reliable measurement of MTJ displacement, which may facilitate further research and applications related to the architectural change of muscles and tendons.

## 1. Introduction

The muscle-tendon unit (MTU) that consists of muscles and tendons plays an important role in force generation and energetics during human movement [1]. The force generated by the contractions of muscle fascicles is transmitted to bones via tendons to control and regulate body motions. The interaction between muscles and tendons takes place in response to the effective utilization of the force and tendon elasticity in mechanical demands during specific movement tasks [2, 3]. Nevertheless, the MTU length calculated using body segment length and joint kinematics is not a good predictor of the muscle and tendon and their interaction [4]. Recently, ultrasound imaging has become a prospective field of research in comprehending the adaptation of muscles and tendons as well as evaluating their function and pathological status by monitoring the architectural changes among muscles and tendons. Sonography has been used to examine muscle-tendon architectural changes in response to motion [2, 3, 5, 6], contraction [7, 8], aging [9, 10], pathologies [11], and physical training [12, 13]. Ultrasound imaging shows great potential in both diagnosis and rehabilitation by assessing geometrical changes in muscles and tendons* in vivo* [14–16].

Changes in tendon length and fascicle length are the most often used structural parameters in ultrasound images to quantify the modulation of interaction between muscles and tendons and estimate the mechanical properties of tendons. Using ultrasound to measure dynamic tendon length changes, the Achilles tendon has been figured out to recoil and stretch in the stance period of walking and take up most of the MTU lengthening [17]. On the other hand, during early stance of walking, the muscle fascicle operates over the highest force region of the force-length curve with the advantage of relatively greater force generation [3, 5]. The rapid shortening of tendon structures has also been found at the first stage of plantar flexion in the fast stretch-shortening cycle exercise, enabling the working muscles to develop more tension in relation to their force-velocity properties [18]. Previous research has pointed out that the mechanical energy generated by the muscle was mainly stored in the tendinous tissue which acts as a spring to store and return elastic energy during human motion [2, 3, 5]. These morphometric parameters are therefore of great importance to understanding muscle-tendon architecture as well as its mechanism. However, the analysis used in most of the previously reported studies relied on the manual method that is time-consuming and potentially prone to human error. Although some automatic approaches have been developed to estimate the muscle fascicle changes [19–29], there is still a lack of methods for measuring the change of tendinous tissues. Moreover, the muscular and tendinous structures are often obscured by speckle noise, making it difficult to measure these morphometric parameters accurately.

**Figure 1:**
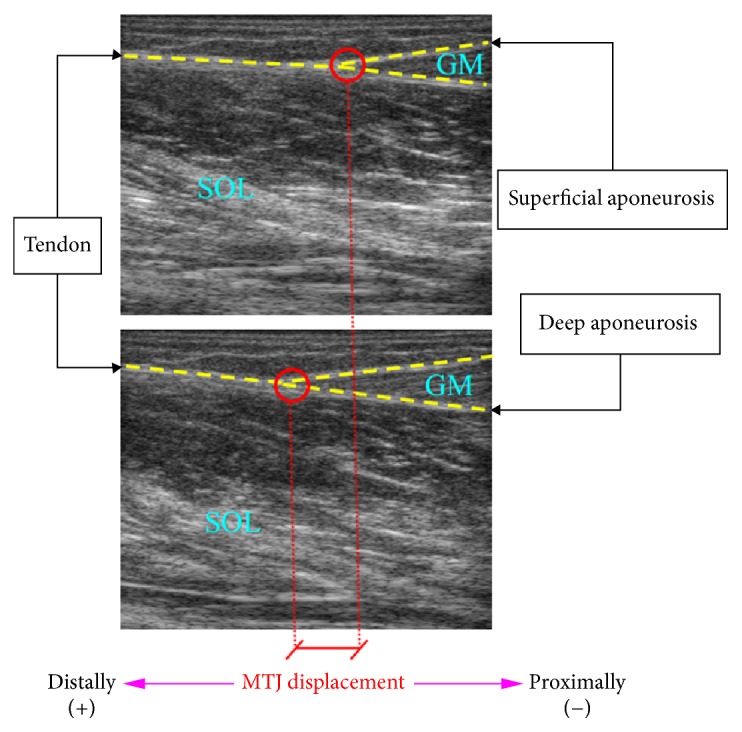
Typical ultrasound image of GM MTJ.

Tendon length changes* in vivo* are usually estimated by tracking the displacement of the myotendinous junction (MTJ) (Figure 1), where the muscle is connected to the tendon, that is, the distal end of the muscle [30]. Motion between sequential images could be applied to quantify the MTJ displacement with the optical flow technique that has been widely used in fields of computer vision such as object segmentation and motion detection [31]. Spatial cross-correlation has been employed to track the tendinous displacement [32]. However, the regions of interest used in the cross-correlation method may undergo irregular changes in appearance or intensity, causing the failure to track the displacement continuously. On the other hand, under the assumption of homogeneous affine transformations in the region of interest, the global affine transformation parameters can be obtained to derive the changes of muscular and tendinous structures using the Lucas-Kanade optical flow algorithm [31]. The changes in fascicle length have been identified with the Lucas-Kanade method by regarding the selected fascicle region as a whole patch [20–22]. Recently, the Lucas-Kanade method was adopted to track the aponeurosis of the rectus femoris for estimating the muscle thickness [24]. Nonetheless, as shown in Figure 1, the motion of different tissues connected in MTJ, including gastrocnemius medialis (GM), gastrocnemius lateralis (GL), soleus (SOL), and Achilles tendon, might violate the assumption of homogeneous affine transformations, resulting in an inaccurate estimation of MTJ displacement when using the Lucas-Kanade method.

In this study, a novel automatic method is presented to estimate the MTJ displacement in consecutive ultrasound images of GM by utilizing prior knowledge on the tendinous tissues and MTJ. Based on the observation that the tendinous tissues are distributed as continuous hyperechoic bands in ultrasound images [33], the symmetric features were first measured with the phase congruency, enabling the segmentation of the effective MTJ region using the localized Radon transform (LRT) and thresholding techniques. Under the assumption of homogeneous affine transformations, the global affine transform parameters are then calculated over the effective MTJ region, addressing the limitations of currently available Lucas-Kanade approaches. The displacement of MTJ was finally estimated by tracking the connection of muscle to Achilles tendon, that is, the intersection of two aponeuroses. The automatic method for MTJ displacement estimation is described in detail in the following section and was tested with real ultrasound image sequences of the GM MTJ during the passive rotation of the ankle joint.

## 2. Materials and Methods

**Figure 2:**
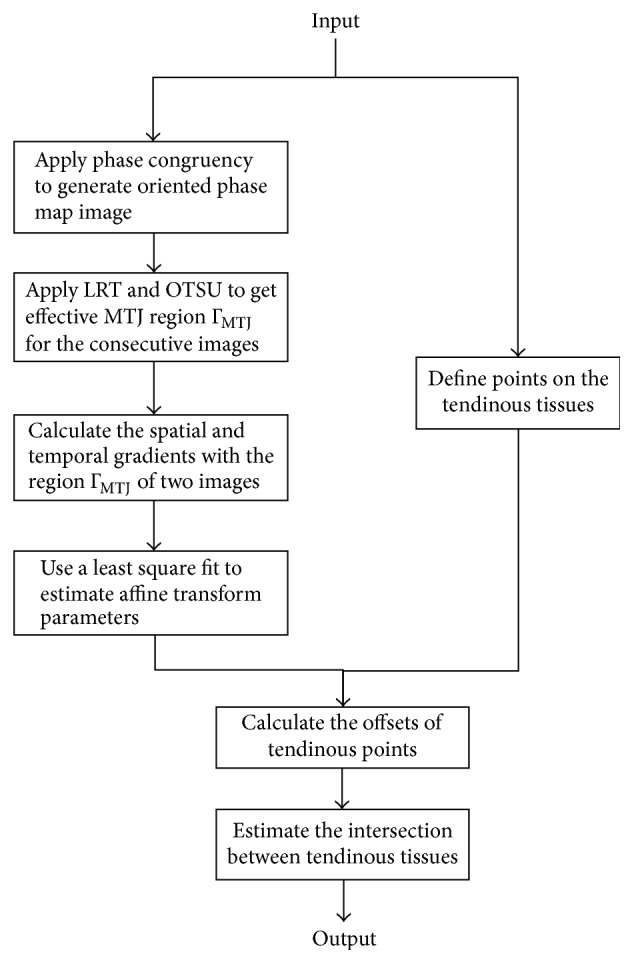
Flowchart of the proposed tracking algorithm.

The flowchart of our proposed method for measuring MTJ is illustrated in Figure 2. The musculoskeletal ultrasound image was first preprocessed with the phase congruency [34] to measure the symmetric features, contributing to the detection of tendinous tissues. As shown in Figure 1, the MTJ was defined as the intersection of two aponeuroses, that is, near the distal end of the muscle. The region containing the visible MTJ structure can then be separated from preprocessed data using the prior anatomical information of MTJ to localize the Radon transform [25] and Otsu method [35]. Under the assumptions of homogeneous affine transformations, the global affine transform parameters were calculated between successive images from the Lucas-Kanade method [36] over the segmented region, termed the effective MTJ region. After the displacements of the predefined points on the tendon and aponeuroses were calculated from the global affine transform parameters, the MTJ was finally determined according to the intersection of two aponeuroses (Figure 1). In cases where the MTJ moved out of the view of ROI, the MTJ was estimated by linearly extrapolating both paths of aponeuroses in ROI.

### 2.1. Preprocessing with Phase Congruency

In musculoskeletal ultrasound images, the tendinous tissues, including aponeuroses and tendons, depict ridge-like hyperechoic bands [33], representing an axis of local symmetry (Figure 1). Phase congruency is a well-known illumination and contrast invariant measure of symmetric features based on the local-energy model [37]. Under the observation that the Fourier series at points of symmetry is either at minima or maxima of their cycle, symmetry in image intensity gives rise to special patterns using phase congruency [34]. After the publication of the phase congruency method [34], it has been investigated extensively to construct descriptors for the ridge-like bone surface localization [38, 39] and cardiac border enhancement [40] in ultrasound images. In this work, we proposed to use the phase congruency as a sensitive feature for the measure of ridge-like tendinous tissues in ultrasound images.

The current state-of-the-art method is to use a quadrature pair of filters to calculate the phase congruency [41–44]. The log Gabor wavelet is the most common choice of quadrature filters because it can achieve good feature localization and noise compensation [41]. By taking the responses of log Gabor wavelet over multiple scales and orientations, the symmetric phase measure at each point in the image can be calculated according to the following equation [41]:

(1) PSx,y=∑r∑sWx,yArsx,yersx,y−orsx,y−Tr∑r∑sArsx,y+ε,

where *e*_*rs*_(*x*, *y*) is the even symmetric part and *o*_*rs*_(*x*, *y*) is the odd symmetric part of the filter at orientation *r* and scale *s*. At a point of symmetry, the absolute value of *e*_*rs*_ will approach 1 and the absolute value of *o*_*rs*_ will be near 0, and vice versa. *ε* is a small real number to avoid division by zero, *T*_*r*_ is the noise threshold, and ⌊·⌋ denotes the operation ⌊*z*⌋ = *z* if *z* > 0; otherwise, ⌊*z*⌋ = 0. *W*(*x*, *y*) is the weighting factor based on frequency spread that reduces phase congruency at locations with a narrow frequency component. *A*_*rs*_(*x*, *y*) is the amplitude of wavelet response with a given scale and orientation at point (*x*, *y*).

**Figure 3:**
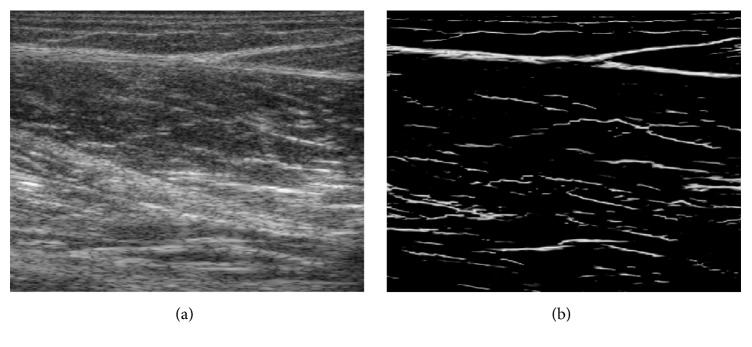
An example showing the oriented phase map: (a) original musculoskeletal ultrasound image contains MTJ; (b) the oriented phase map of the image.

Moreover, the angular range of tendinous tissues has been reported to be about −10° to 10° [45]. Phase congruency with specified central orientation, called oriented phase congruency, could be used to further measure ridge-like tendinous tissues, which is calculated as follows:

(2) $$\begin{array}{c} {PS}_{Orient}\left(\;x,y\;\right)=\frac{\sum_{s}W\left(x,y\right)\left\lfloor A_{r_{spec}s}\left(x,y\right)\left[\left|e_{r_{spec}s}\left(x,y\right)\right|-\left|o_{r_{spec}s}\left(x,y\right)\right|\right]-T_{r_{spec}}\right\rfloor }{\sum_{s}A_{r_{spec}}\left(x,y\right)+\varepsilon }, \end{array}$$

where *r*_spec_ is the specified orientation range covering [−10°, 10°]. The parameters used in computing phase congruency, such as *W*(*x*, *y*), *A*_*rs*_(*x*, *y*), and *T*_*r*_, were based on those presented in [41]. Figures 3(a) and 3(b) show an example of an original image and its corresponding oriented phase map, respectively.

### 2.2. Effective MTJ Region Segmentation

Both aponeuroses and fascicles are distributed as line-like structures in musculoskeletal ultrasound images [19, 20]. The oriented phase congruency thus enhanced the ridge-like features of not only tendinous tissue but also part of fascicles in GM and SOL muscles. Nevertheless, the tendinous tissues are represented as continuous hyperechoic bands in the ultrasound image [33], while the fascicles are usually not uniformly distributed as line-like structures [46], which makes the depiction of tendinous tissue quite distinct from that of fascicles in the oriented phase map (Figure 3(b)). Moreover, the MTJ is the site of connection between tendons and muscles. As illustrated in Figure 1, the MTJ was observed to be the intersection of two aponeuroses, being used to localize the Radon transform over the oriented phase map. Motivated by the aforementioned observation on tendinous tissues and MTJ, the LRT was employed to roughly determine the MTJ region from the oriented phase map [25]. The LRT over Euclidean space is defined as

(3) RLOCθ,ρ=∫xminxmax∫yminymaxPSOrientx,y·δρ−xcos⁡θ−ysin⁡θdx dy,

where PS_Orient_(*x*, *y*) is the oriented phase map at position (*x*, *y*) and *δ* is the Dirac delta function. *ρ* and *θ* denote the distance from the center of the image to the straight line and the angle between the *x*-axis and the line perpendicular to the straight line, respectively. Only the points *x*_min_ ≤ *x* ≤ *x*_max_ and *y*_min_ ≤ *y* ≤ *y*_max_ in image space and *θ*_min_ ≤ *θ* ≤ *θ*_max_ in Radon space are calculated in LRT. The same revoting strategy [47] was conducted to extract line features one by one and remove all phase maps close to the detected lines. According to a previous study [45], the angular range was set to be −10° ≤ *θ* ≤ 10° for performing LRT. In addition, based on the prior knowledge on MTJ structure (Figure 1), the position and orientation of the detected lines were used to determine the proper range of the locations and the orientation where other aponeuroses and tendons are supposed to be found. Moreover, the average diameter of tendinous tissues of GM tendon and aponeuroses has been reported to be about 2.5 mm [48]. The removing width was then empirically determined to be 2 mm in this study.

**Figure 4:**
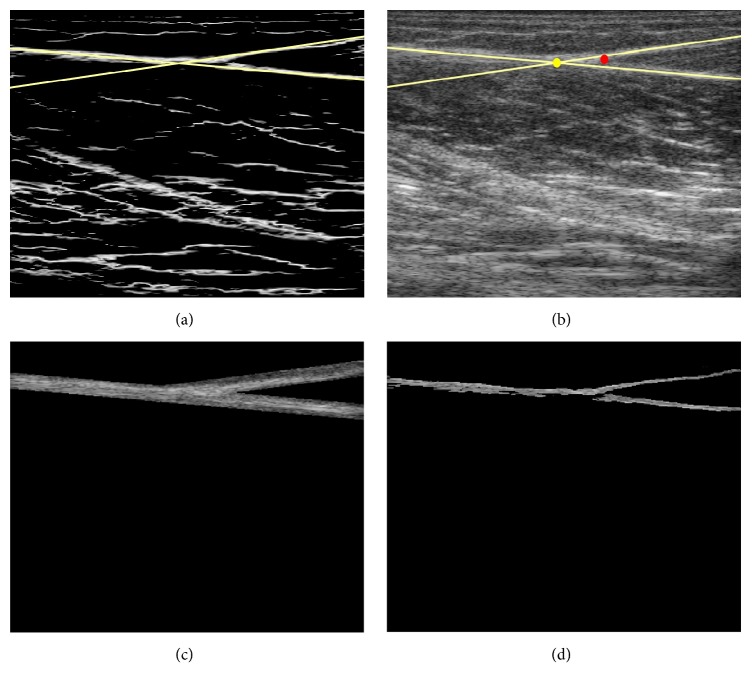
An example of effective MTJ region segmentation from the image shown in Figure 3: (a) the LRT results on the oriented phase map shown in Figure 3(b); (b) the intersection of LRT on the image shown in Figure 3(a); (c) the result of the tendinous region; (d) the effective MTJ region segmentation result. Yellow lines are the lines detected by LRT on the oriented phase map. The circles of yellow and red represent the MTJ identified by the intersections of LRT and manual measurements, respectively.

After localization and revoting procedure, lines intersecting near MTJ could be identified from the Radon space obtained from the oriented phase map.  Figure 4(a) presents an example of LRT on the oriented phase map as shown in Figure 3(b). However, a common limitation of phase-based techniques is the poor localization on blurred features, affecting the localization of detected lines and MTJ. As illustrated in Figure 4(b), the intersection determined by the lines from the phase map was not well located at the actual MTJ. Therefore, in this study, instead of directly locating the MTJ by LRT, the effective region of MTJ was segmented from the oriented phase map by combining the LRT and Otsu method, enabling the reliable tracking of MTJ with the Lucas-Kanade method. Based on previously reported average diameters of tendinous tissues [48], a distance threshold *T*_*d*_ to the detected lines was firstly set to 2.5 mm to exclude the nontendinous region and determine the tendinous region Γ_T_. The Otsu method was then employed to calculate the global threshold *T*_Otsu_ on the oriented phase map over the tendinous region Γ_T_. The final effective MTJ region Γ_MTJ_(*x*, *y*) was derived as follows:

(4) ΓMTJx,y=1,x,y∈ΓT,  PSOrientx,y≤TOtsu0,otherwise.

Figures 4(c) and 4(d) illustrate the tendinous region Γ_T_ and the corresponding effective MTJ region Γ_MTJ_ on the ultrasound image (Figure 3(a)), respectively.

### 2.3. Automatic MTJ Tracking Using Lucas-Kanade Optical Flow Method

The MTJ could be identified as the intersection of two aponeuroses near the distal end of the muscle (Figure 1). The points on the aponeuroses were used to determine the position of MTJ, which could be manually defined by identifying the aponeuroses skeleton in the first frame, and then tracked using the Lucas-Kanade method for the subsequent images. Let  *I*(*x*, *y*) denote the gray-level value at pixel  (*x*, *y*); the image constraint equation between time *t* and *t* + Δ*t* is

(5) $$\begin{array}{c} I\left(\;x,y,t\;\right)=I\left(\;x+\Delta x,y+\Delta y,t+\Delta t\;\right). \end{array}$$

With the assumption of small movement between adjacent images, the image constraint at *I*(*x*, *y*, *t*) with Taylor series can be expressed as

(6) Ix+Δx,y+Δy,t+Δt=Ix,y,t+∂I∂xΔx+∂I∂yΔy+∂I∂tΔt+ε,

where *ε* is the higher-order term. It follows that

(7) $$\begin{array}{c} \frac{\partial I}{\partial x}\Delta x+\frac{\partial I}{\partial y}\Delta y+\frac{\partial I}{\partial t}\Delta t=I_{x}V_{x}+I_{y}V_{y}+I_{t}V_{t}=0, \end{array}$$

where *V*_*x*_ and *V*_*y*_ are the *x* and *y* components of the velocity or optical flow of *I*(*x*, *y*, *t*) and *I*_*x*_, *I*_*y*_, and *I*_*t*_ are the derivatives of *I* with respect to *x*, *y*, and *t*. In Lucas-Kanade optical flow method [21], the image velocity is defined by six affine transform parameters:

(8) $$\begin{array}{c} \left(\;V_{x},V_{y}\;\right)=\left[\begin{array}{ccc} x & y & 1 \end{array}\right]\times \left[\begin{array}{cc} d+s1 & s2+r\\ s2-r & d-s1\\ vxt & vyt \end{array}\right], \end{array}$$

where the affine flow parameters *vxt* and *vyt* are the optical flow at the origin in the *x*- and *y*-directions, respectively, *d* is the rate of dilation, *r* is the rate of rotation, *s*1 is the shear along the main image axis, and *s*2 is the shear along the diagonal axis.

Under the assumption of homogeneous affine transformations, the six affine transform parameters could be determined from a least square fit using the given spatial and temporal gradients over the whole region between adjacent images [31]. The *x* and *y* grids along with the spatial and temporal gradients were resampled (every 3 pixels) to reduce the computation cost in the least square fit [21]. However, the visible nontendinous components and speckle noise in the selected region were not taken into account in this resampling method, which may violate the hypothesis of homogeneous affine transformations in the whole selected region, resulting in the inaccurate estimation of the global affine flow parameters. Therefore, we proposed a simple and automatic method to calculate the affine transform parameters over the effective MTJ region Γ_MTJ_ obtained using the prior information of MTJ, to achieve both reliable calculation and economical computation cost for the least square fitting process. Only the spatial and temporal gradients in Γ_MTJ_ were applied to calculate the affine transform parameters via a least square fit, which could reduce the error caused by the nontendinous components and speckle noises. The predefined points were then applied to determine the position of aponeuroses by calculating their displacements from the affine transform parameters. Finally, according to the definition of MTJ (Figure 1), the intersection of two aponeuroses was calculated to measure the displacement of MTJ. In cases where the MTJ moved out of the view of ROI, the MTJ was estimated by linearly extrapolating both paths of aponeuroses in ROI.

### 2.4. Experiment

Ten healthy adults (age: 26.0 ± 2.9 years; 6 males and 4 females; weight: 70.4 ± 11.5 kg; body mass index: 22.8 ± 2.2 kg/m^2^) with no history of musculoskeletal injury were recruited in an experimental study to demonstrate the feasibility of the proposed method. This study was approved by the Regional Ethics Committee, Stockholm, Sweden. All subjects signed informed consent before participation in the study.

**Figure 5:**
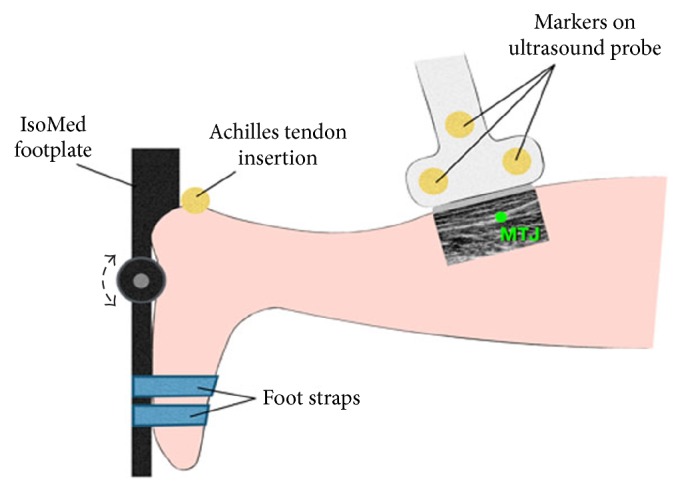
Illustration of the experimental setup during the passive rotation test of the ankle joint.

The subjects were instructed to lie in a prone position with their knee flexed at 20° and their foot fixated to a footplate connected to a dynamometer (Figure 5). Only the right foot was tested in the convenience of the experimental setup. Shoulders, hips, legs, and the tested foot were adequately fixated, while paying special attention to securely strapping the foot to the footplate. The ankle joint was carefully aligned with the dynamometer axis of rotation using a laser device. In the initial position, the footplate was positioned perpendicularly to the tibia of the subject, which was defined as 0° ankle rotation. In the following, plantar flexion of the foot will be expressed in negative (−) angles, and dorsiflexion of the foot will be expressed in positive (+) angles. The ankle of all participants was passively rotated between −20° and 10° several times to familiarize the movement. For the actual experiment, the ankle was rotated five consecutive times at a constant velocity of 5°/s within the range of motion (ROM). All participants were instructed to stay relaxed during the passive rotation experiments.

During the passive ankle rotation, the excursion of the MTJ of GM was recorded using an ultrasonography system, while the corresponding ankle angle was recorded by the dynamometer at 5 kHz. The position of MTJ at 0° ankle rotation was selected for the initial position to calculate MTJ displacement. The positive (+) displacement expresses that the MTJ moves distally during dorsiflexion and vice versa (Figure 1). An ultrasound scanner (Vivid Q, GE Healthcare, Milwaukee, WI, USA) with a linear transducer was utilized to capture MTJ excursion, which was sampled at 40 frames/s with an image resolution of 0.11 mm/pixel. The ultrasound transducer probe with a frequency of 3.5–10 MHz and a field of view of 53 mm was optimally positioned parallel to the tendon in the sagittal plane, and the ultrasound image plane was therefore aligned with the longitudinal axis of the tendon. The MTJ displacement was automatically estimated with our proposed approach developed using Visual Studio (Microsoft Corporation, Washington, USA) in the present study. To evaluate the performance of our proposed method, the traditional Lucas-Kanade method [36] was also applied to track the MTJ displacement. Moreover, manual measurements of MTJ displacement were performed three times in each image by a single expert who was experienced in ultrasound imaging of muscles and blind to the automatic measurement results. According to the definition of MTJ displacement, line segments representing tendinous tissues were manually drawn in each ultrasound image to obtain an estimate of MTJ and its movement. The mean value of the manual displacement measurements was used to compare with the automatic measurements.

### 2.5. Data Analysis

Values were reported as mean (±SD) for all subjects unless otherwise stated. The manual measurements of MTJ displacement were used as a reference for comparison with the automatic measurements. The intraclass correlation coefficient (ICC) was used to test the intraobserver repeatability of manual measurement [49]. The waveform similarity between the manual and automatic measurements was assessed by calculating the overall similarity with the coefficient of multiple correlations (CMC) [50, 51], with a range of 0 and 1. More similar waveforms have higher CMC values, whereas highly dissimilar waveforms can result in a CMC near 0. Student's paired *t*-test was applied to test the difference between the CMC values of the proposed method and the Lucas-Kanade method. Moreover, Bland and Altman's method of differences [52] was applied to test the agreement between the manual and automatic measurements. In addition, polynomial regression analysis was applied to describe the association between the ankle angle rotation and MTJ displacement. Pearson's product-moment correlation (*r*) was calculated for the regression analysis. The level of significance was accepted at *p* < 0.05.

## 3. Results

**Figure 6:**
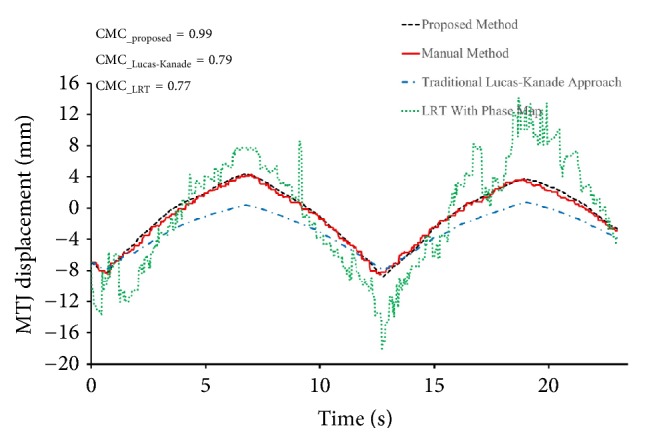
The MTJ displacement obtained with the proposed method, the traditional Lucas-Kanade approach, the LRT with phase map, and the manual method during the passive rotation test of the ankle joint for subject H.

**Figure 7:**
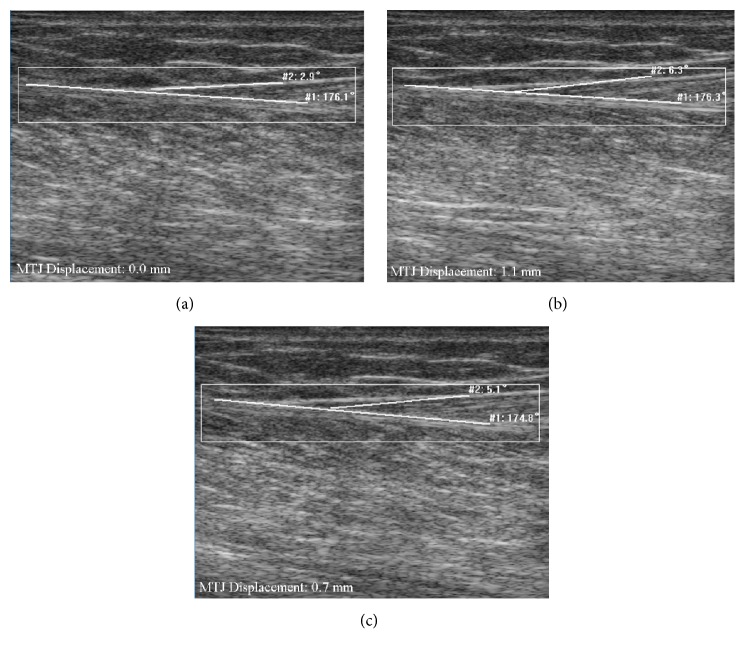
The measurement of MTJ displacement for subject H using traditional Lucas-Kanade approach: (a) the MTJ in the 1st frame manually defined with the lines (white line); (b) the measurement of MTJ at frame 275; (c) the measurement of MTJ at frame 764.

**Figure 8:**
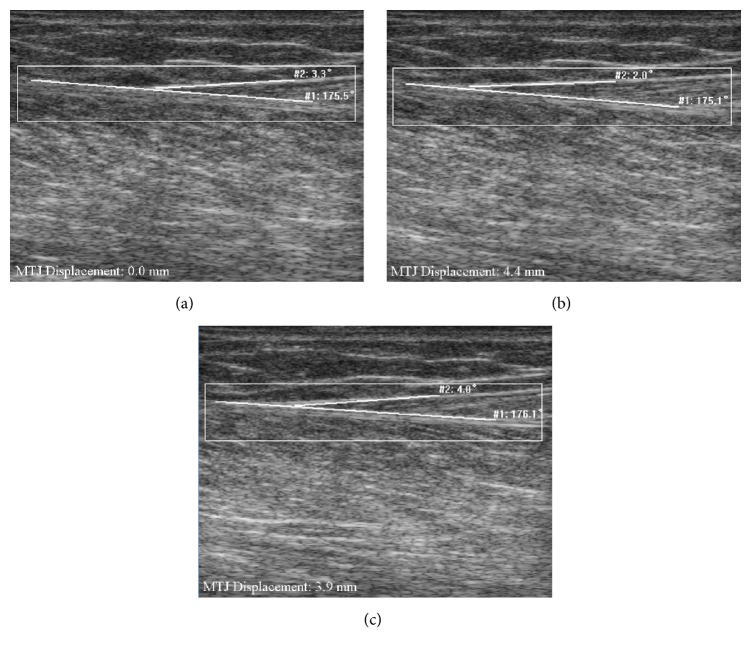
The measurement of MTJ displacement for subject H using the proposed approach: (a) the MTJ in the 1st frame manually defined with the lines (white line); (b) the measurement of MTJ at frame 275; (c) the measurement of MTJ at frame 764.

**Figure 9:**
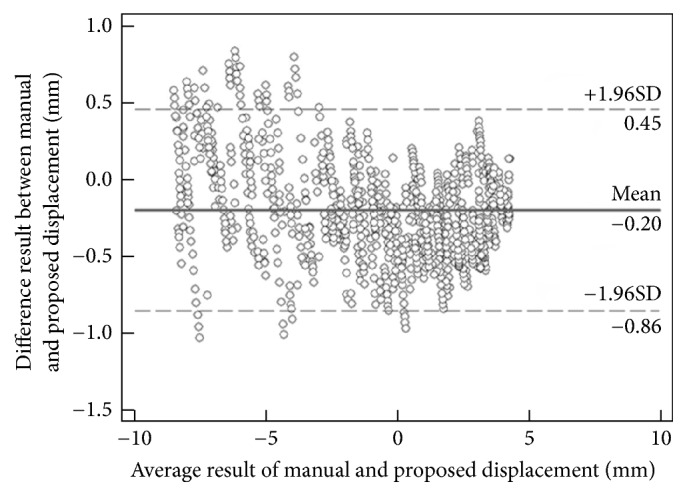
Bland-Altman plot of the MTJ displacement measured with the proposed method and manual measurements.

As shown in Table 1, the CMC value (0.79 ± 0.11) determined by the Lucas-Kanade method ranged from 0.57 and 0.95, while the proposed method had a CMC value (0.97 ± 0.02) ranging from 0.94 to 0.99. The difference in the CMC value between these two approaches was significant (*p* < 0.05).  Figure 6 shows a typical example of MTJ displacement measured with the proposed approach, for which Lucas-Kanade method failed to track the excursion in MTJ. Since the tendinous structures in the image were not taken into account, such errors could not be avoided when the whole selected region was used to estimate the affine transform parameters (Figures 7(b) and 7(c)). On the other hand, our proposed tracking approach only used the effective tendinous region to calculate the affine transform parameters, thus avoiding the influence of nontendinous components and speckle noise (Figures 8(b) and 8(c)). The CMC values also suggested that the automatic measurement with the proposed method was more consistent with the manual measurement compared with the Lucas-Kanade method.  Figure 6 also illustrated that the poor localization of phase-based techniques degraded the accuracy of MTJ localization when directly using LRT to detect the MTJ on the oriented phase map. As shown in Figure 9, the Bland-Altman plot between the manual and automatic measurements indicated a low mean difference (0.2 mm) and the symmetrically distributed difference around mean difference was within limits (±1.96 SD = 0.65 mm), suggesting a good agreement between the measurements obtained by our proposed method and the manual method. Additionally, the intraobserver tests of manual MTJ measurements showed good repeatability, with the ICC being 0.91 ± 0.03 (*p* < 0.001). These results support the conclusion that the proposed approach was reliable for the estimation of MTJ displacement.

**Figure 10:**
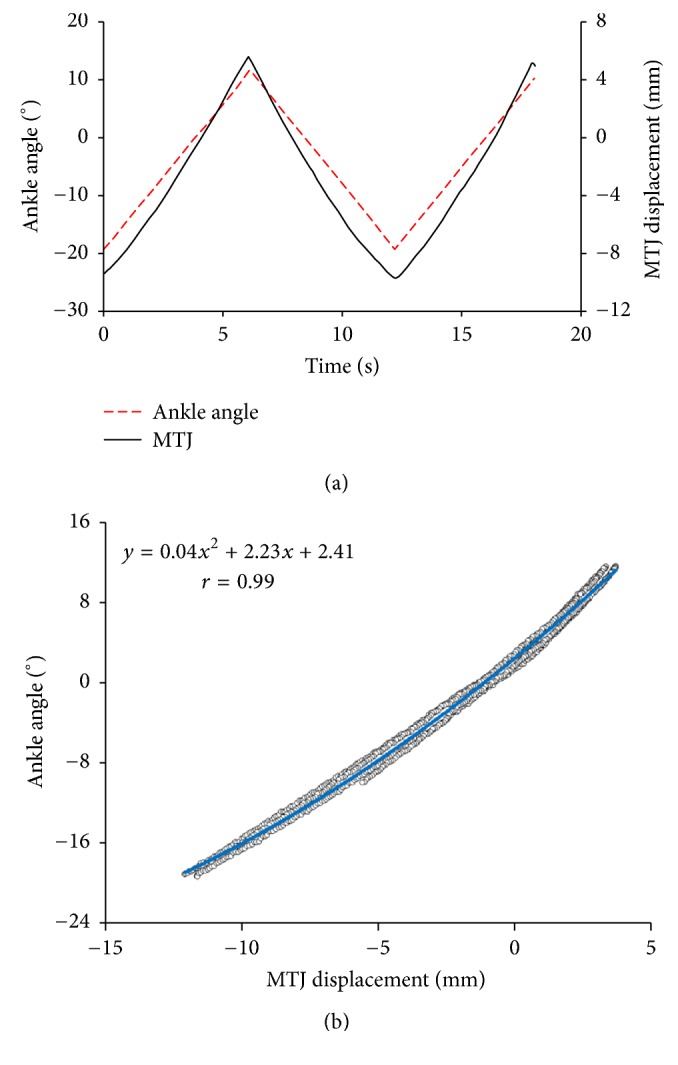
The displacement of MTJ during the passive rotation test of the ankle joint for subject A: (a) the change of MTJ and ankle angle with time; (b) the cross-correlation between MTJ displacement and ankle angle.

The MTJ displacement shifted nonlinearly with the change of ankle angle during passive ankle rotation.  Figure 10 demonstrates the changes MTJ with the change in ankle angle for one typical subject. When the ankle angle was rotated from −19.26 ± 0.06° to 11.64 ± 0.05°, the MTJ measured with the proposed method was moved from −8.22 ± 2.03 mm (proximally) to 3.97 ± 1.27 mm (distally) and was correlated with the ankle joint angle (*r* = 0.99 ± 0.01, all *p* < 0.001). Also, the average computation time for the estimation of fascicle length with the proposed method in the ~400 × 400 pixel region was about 2~3 sec for each frame using a computer with an Intel Core 7 2.60 GHz processor and 4 GB of memory.

## 4. Discussion

We have developed a novel automatic method to track the MTJ displacement in sequential musculoskeletal ultrasound images. The combination of phase congruency with LRT made it feasible to segment the effective MTJ region from ultrasound images using the prior knowledge of MTJ structure. A more reliable calculation of global affine transform parameters was then achieved using the Lucas-Kanade optical flow algorithm over the effective MTJ region since it precluded the influence of nontendinous components as well as speckle noise on the motion estimation of points on the tendinous tissues. The* in vivo* experiment results show that MTJ could be reliably tracked in continuous ultrasound images, which were in good agreement with those obtained by manual measurement and correlated well with kinematic data, such as ankle angle.

The key element in the proposed method is to utilize prior knowledge of MTJ structures in the musculoskeletal ultrasound images. Tendons are made up of collagen fibers and flattened wide tendons are known as aponeuroses that are often found in series with a tendon [33, 53]. Both tendons and aponeuroses are distributed as continuous hyperechoic bands with specified orientation in the ultrasound image [33, 45], representing an axis of local symmetry. As an illumination and contrast invariant measure of symmetric structures, phase congruency can be employed to perceive and enhance ridge-like features [34], which is beneficial for the detection of tendinous tissues using Radon transform. Moreover, MTJ is the specific site of connection between tendons and muscles [54], which can be identified as the intersection of two aponeuroses in ultrasound images [55, 56]. With the consideration of the poor localization of phase-based techniques, the locations and the orientation of tendinous tissues and MTJ were used to facilitate the segmentation of MTJ obtained with LRT and Otsu methods. The prior knowledge of MTJ structures, therefore, allows for easier detection of the effective MTJ region from the musculoskeletal ultrasound images, which can be applied as a preprocessing step for the tracking of MTJ displacement.

The estimation of affine flow parameters over the effective MTJ region could avoid the accumulation of errors caused by inhomogeneous deformation across the area of interest between consecutive images. The proposed method segments visible MTJ structures from the whole image for the calculation of global affine transform parameters, thus obviating the impact the nontendinous components and speckle noise. On the other hand, the original Lucas-Kanade method with the whole region, including MTJ structure, nontendinous components, and speckle noise, might cause the inaccurate estimation of affine transform parameters, resulting in accumulated measurement errors of MTJ displacement. The overall high CMC value (0.97 ± 0.02) demonstrated that the results of the proposed method were more consistent with the manual than that of the original Lucas-Kanade method (0.79 ± 0.11). The significantly larger CMC value for the proposed method (*p* < 0.05), compared with the Lucas-Kanade method, also suggested that this approach had a better performance in tracking MTJ displacement.

In this study, the average excursion of MTJ was −8.22 ± 2.03 mm with the plantar flexion angle reaching 19.26 ± 0.06°, and MTJ was moved distally by 3.97 ± 1.27 mm for an ankle angle of 11.64 ± 0.05°. These results were in line with those reported in previous work [56, 57]. The average displacement of MTJ was reported to be 14 mm from 20° plantar flexion to 10° dorsiflexion [56]. Additionally, the greater displacement in the females reported in [57] was also found in our study, implying lower muscle stiffness in females than in males [57, 58]. In the previous study of muscles and tendons, the changes in tendon length can be obtained by subtracting MTJ displacement from MTU length change estimated using ankle joint angle [59]. Therefore, it is feasible to generalize the use of MTJ displacement into tendon length change analysis with the proposed method, which would facilitate an improved understanding of the structural and bioelectrical properties of muscles and tendons during motion.

However, the method proposed in this study still had some limitations. Firstly, errors might be introduced if the assumption that tendinous tissues being tracked conform to homogeneous affine transformations does not hold. Given the promising results, it seems that this assumption holds true when tracking the planar movement of tendinous tissues during passive motion. Further studies should be conducted to take account of both changes in local shape and global shape, thus improving the tracking accuracy of MTJ. In addition, manual initialization of points on the tendinous tissues might also affect the results of MTJ estimation. The automatic initialization should be further investigated in future studies with better detection of line and corner features in musculoskeletal ultrasound images. Furthermore, a two-point representation may not properly account for the Achilles tendon curvature. The Achilles tendon is almost straight in the dorsiflexion region; as the ankle joint angle changes, the Achilles tendon becomes slightly curved, resulting in an approximately 3% underestimation of tendon length with the plantar flexion angle reaching 30° [55]. Thus, this effect is likely to be a minor factor in measuring MTJ and tendon length in daily human movements. Nevertheless, it would be ideal if the algorithm was able to track multiple points along a tendinous tissue for curvature measurement in the future.

## 5. Conclusions

We have successfully developed a robust method for automatically tracking MTJ displacement in a series of GM ultrasound images with the prior knowledge about the distribution and shape of tendinous tissues and MTJ. The proposed method, therefore, precluded the influence of nontendinous components on the calculation of affine transform parameters over effective MTJ region segmented by combining phase congruency with LRT and thresholding methods, resulting in a more reliable estimation of MTJ displacement. The results showed a good agreement between the automatic and manual measurements. This approach obviated subjective manual measurements, reducing the variations in measurements. We expected that the proposed method would provide an effective way to analyze the functionality of muscle-tendon unit in human kinetics as well as force generation analysis. Future studies with a large cohort of subjects, including patients with musculoskeletal abnormalities, will be conducted to further illustrate the potential of this new approach for the full understanding of muscle as well as tendon mechanism. The performance of the newly proposed method can also be further enhanced by taking account of both changes in local shape and global shape in future studies.

## Figures and Tables

**Table 1:** The CMC values between the manual and automatic methods for the measurement of MTJ displacement.

Subject	CMC value between manual measurement and automatic measurement by the Lucas-Kanade method	CMC value between manual measurement and automatic measurement by the proposed method
A	0.88	0.97
B	0.91	0.99
C	0.57	0.94
D	0.72	0.96
E	0.73	0.97
F	0.95	0.96
G	0.87	0.97
H	0.79	0.99
I	0.70	0.94
J	0.78	0.97

## Supplementary Materials

Supplementary Materials The Supplementary Materials include one sample video showing the MTJ tracking results obtained by our proposed method.
